# Real-time modulation of visual feedback on human full-body movements in a virtual mirror: development and proof-of-concept

**DOI:** 10.1186/1743-0003-12-2

**Published:** 2015-01-05

**Authors:** Meyke Roosink, Nicolas Robitaille, Bradford J McFadyen, Luc J Hébert, Philip L Jackson, Laurent J Bouyer, Catherine Mercier

**Affiliations:** Centre Interdisciplinaire de Recherche en Réadaptation et Intégration Sociale (CIRRIS), 525 Boul Hamel, Québec, QC G1M 2S8 Canada; Department of Rehabilitation, Faculty of Medicine, Laval University, Québec, QC Canada; Canadian Forces Health Services Headquarters, Directorate of Medical Policy (Physiotherapy), Valcartier Garrison, Québec, QC Canada; Department of Radiology, Faculty of Medicine, Laval University, Québec, QC Canada; School of Psychology, Laval University, Québec, QC Canada

**Keywords:** Motion capture, Visual feedback, Proprioception, Physical rehabilitation, Virtual reality, Body perception

## Abstract

**Background:**

Virtual reality (VR) provides interactive multimodal sensory stimuli and biofeedback, and can be a powerful tool for physical and cognitive rehabilitation. However, existing systems have generally not implemented realistic full-body avatars and/or a scaling of visual movement feedback. We developed a “virtual mirror” that displays a realistic full-body avatar that responds to full-body movements in all movement planes in real-time, and that allows for the scaling of visual feedback on movements in real-time. The primary objective of this proof-of-concept study was to assess the ability of healthy subjects to detect scaled feedback on trunk flexion movements.

**Methods:**

The “virtual mirror” was developed by integrating motion capture, virtual reality and projection systems. A protocol was developed to provide both augmented and reduced feedback on trunk flexion movements while sitting and standing. The task required reliance on both visual and proprioceptive feedback. The ability to detect scaled feedback was assessed in healthy subjects (n = 10) using a two-alternative forced choice paradigm. Additionally, immersion in the VR environment and task adherence (flexion angles, velocity, and fluency) were assessed.

**Results:**

The ability to detect scaled feedback could be modelled using a sigmoid curve with a high goodness of fit (R^2^ range 89-98%). The point of subjective equivalence was not significantly different from 0 (i.e. not shifted), indicating an unbiased perception. The just noticeable difference was 0.035 ± 0.007, indicating that subjects were able to discriminate different scaling levels consistently. VR immersion was reported to be good, despite some perceived delays between movements and VR projections. Movement kinematic analysis confirmed task adherence.

**Conclusions:**

The new “virtual mirror” extends existing VR systems for motor and pain rehabilitation by enabling the use of realistic full-body avatars and scaled feedback. Proof-of-concept was demonstrated for the assessment of body perception during active movement in healthy controls. The next step will be to apply this system to assessment of body perception disturbances in patients with chronic pain.

## Background

The normalization of body perception disturbances and of abnormal movement patterns is an important goal in both physical and pain rehabilitation. This requires an understanding of the complex relationship between body perception and movement kinematics, which can subsequently be used to guide patients towards more optimal movement patterns, i.e. by providing visual, haptic and verbal feedback. Virtual reality (VR) is a tool that can create credible and complex multimodal sensory stimuli and biofeedback [1, 2], can increase therapy engagement [3–5], and may distract from effort and pain [5, 6]. Moreover, VR can create visual illusions that “bend the truth”, which could be used to assess or change body perception or to stimulate more optimal movement patterns. Lastly, by combining VR with other technologies such as motion capture, therapies may be better tailored to the individual needs of patients. As such VR has increasingly been explored in the context of rehabilitation. Common applications of VR in rehabilitation include for example self-displacements or object displacements in realistic [7] or non-realistic [8, 9] virtual (gaming) environments, or the manipulation of virtual body parts, e.g. to replace a missing limb in amputee patients [10–12].

Although used extensively in gaming and video-animation, the use of full-body avatars is still rare in rehabilitation due to the need for accurate movement representations requiring detailed movement sampling and modelling, which can be complex and time-consuming. One of the few successful examples is a study by Koritnik et al. who created a full-body “virtual mirror” by recording kinematic data to animate a virtual mirror-image (non-realistic avatar) in real-time, while healthy adults were stepping in place [13]. Another example is a very recent study by Barton and colleagues that implemented a virtual mirror for amputee patients. In their study, movements kinematics of the unimpaired leg were combined with the movement timing of the impaired leg to model a realistic avatar with a symmetric gait pattern [14]. In addition, some studies have used full-body video-capture to display a full-body mirror-image [3, 15] or avatar [16] of the subject onto a virtual reality scene.

Unfortunately, the VR systems commonly available in rehabilitation have some important limitations. The modelling of virtual limbs and avatars has generally been based on specific movements in a limited number of movement planes, whereas rehabilitation may include complex movements in multiple movement planes. In addition, only a few VR systems allow for a scaling of movements (e.g. providing augmented or reduced feedback). Indeed, an altered perception of body movements [17, 18] or body size [19, 20] could be used to promote or prevent certain movement patterns and could directly impact on pain perception [21–23]. For example, previous work has shown that a virtual environment in which movements were scaled to attain reduced movement perception increased the range of neck motion in patients with neck pain as opposed to a virtual environment without scaling [17]. Likewise, a gradual modulation of visual feedback of step-length during gait (simple bar graphs) systematically modulated step length away from symmetry, even when subjects were explicitly instructed to maintain a symmetric gait pattern [18]. However, the required level (low, high) and direction (reduction, augmentation) of scaling is likely to depend on the particular body part and movement involved as well as on the particular type of feedback provided.

As such, and prior to the development of any intervention protocols, it is important to establish normative data regarding body perception during active movement in VR, for example by assessing the ability to detect different levels and directions of scaled feedback in healthy subjects [18]. To attain this goal we developed a “virtual mirror” that: 1) displays a realistic full-body avatar, 2) responds to full-body movements in all movement planes in real-time, and that 3) allows for the scaling of visual feedback on movements at any given joint in real-time.

The primary objective of this proof-of-concept study was to assess the ability of healthy adults to detect scaled feedback on trunk movements using a two-alternative forced choice paradigm. For each subject, a psychophysical curve was created, and two main variables of interest were derived, the point of subjective equality (PSE) and the just noticeable difference (JND). It was expected that healthy adults would perform consistent with expectations for a two-alternative forced choice paradigm, i.e. that the detection of scaled feedback could be modelled using a sigmoid curve, and that subjects would display unbiased perception (no shift in PSE) and high discriminative ability (small JND). Secondary objectives were to assess virtual reality immersion and task adherence (movement kinematics).

## Technological development of the virtual mirror

**Figure 1:**
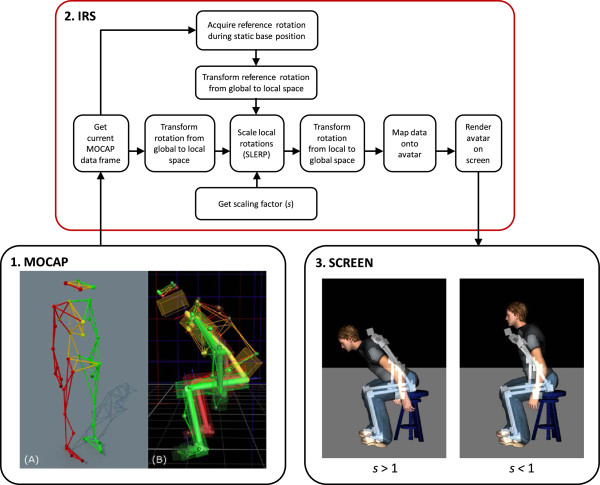
**Overview of the different components of the virtual mirror.** 1) Motion capture (MOCAP) system including the positioning of 41 reflective markers on the subject’s body **(A)** to create a Vicon skeleton template **(B)**; 2) Interaction and rendering system (IRS) that retrieves and scales the MOCAP data online, maps the modified data onto the avatar and renders the avatar on screen; 3) Projection screen displaying the avatar’s movements as being augmented (left, scaling factor *s* > 1) or reduced (right, scaling factor *s* < 1) as opposed to the subject’s actual movements (here displayed as a white skeleton).

The virtual mirror consists of three main components: 1) a motion capture (MOCAP) system, 2) an interaction and rendering system (IRS), and 3) a projection system, see Figure 1. The subject’s movements (rotation and position) are first acquired using the MOCAP system. The data is then sent to the IRS, which scales the subject’s movements and applies the scaled data to an avatar in real-time. The IRS finally displays the avatar onto a projection screen. A mirrored projection setup allows the subjects to see their avatar as a mirror-image.

### Motion capture system

The MOCAP system (Vicon Motion Systems Ltd., Oxford, UK) is used to acquire the subject’s movements, which are then mapped to an avatar in real-time by the IRS. The system consists of 12 infrared cameras (Bonita 10) connected to a computer (Intel Xeon E31270, 3.40 GHz; 4 GB RAM; OS: Windows 7, 64 bits; NVIDIA Quadro 2000) running Vicon’s Nexus 1.8.2 acquisition software. Movements are captured with a sampling frequency of 100 Hz using a set of 41 reflective markers (14 mm) placed on the subject’s entire body. To be able to locate a marker in 3D space, the MOCAP system must be calibrated. The calibration consists of environment reflection removal, a calibration of the cameras using a wand with a specific marker configuration, and setting the volume origin.

The placement of the markers on the subject’s body is facilitated by using a motion capture suit, and is determined by a skeleton template file based on Vicon’s ‘HumanRTkm’ model. This model additionally defines a hierarchy of segments (or bones) consisting of 19 segments. A complete list of segments and their hierarchy is presented in Table 1, and a visual representation is presented in Figure 1 (frame 1). The segments are mapped onto the subject based on another calibration procedure. This procedure consists of 1) acquiring a sequence of predefined body movements of the head, shoulders, arms, trunk and legs; 2) labeling the markers of the acquired sequence according to the skeleton template; and 3) calibrating the position and orientation of the skeleton joints based on the sequence movements. Once the subject is calibrated, real-time segment positions and orientations are transmitted to the IRS through a local network.

**Table 1:** **Motion capture: skeleton segments and hierarchy**

Root segment	Level 1	Level 2	Level 3	Level 4	Level 5
Pelvis	Thorax	Head			
		Clavicle_L	Humerus_L	Radius_L	Hand_L
		Clavicle_R	Humerus_R	Radius_R	Hand_R
	Femur_L	Tibia_L	Foot_L	Toes_L	
	Femur_R	Tibia_R	Foot_R	Toes_R	

L: left, R: right.

### Interaction and rendering system

The IRS consists of a computer (Intel Xeon E31270, 3.40 GHz; 4 GB RAM; Windows 7, 32 bits; NVIDIA Quadro 2000) running D-Flow (Motek Medical, Amsterdam, The Netherlands). The computer receives the MOCAP data, performs the scaling (see paragraph on ‘Movement scaling’ for details) and maps the resulting data onto the avatar rig so that the avatar follows the subject’s movements in real-time at a refresh rate of 60 Hz. A realistic male avatar model was bought (http://www.TurboSquid.com, Martin T-pose, ID 523309) and rigged for motion capture using Blender (Blender Foundation, Amsterdam, The Netherlands). The avatar model was then converted to the OGRE format (Object-Oriented Graphics Rendering Engine, ogre3d.org) to be used in real-time in the IRS. As such, the size and proportions of the avatar vary based on individual MOCAP data whereas its appearance (e.g. body shape, clothing) remains the same for each subject. In principle, the avatar is placed in an empty scene (grey floor, black walls). However, a height-adjustable stool that is present in the laboratory was also modeled in VR and can additionally be presented and adjusted (i.e. height, positioning in the scene) using an interface programmed in D-flow.

### Projection system

The avatar model is projected onto a silver-coated screen (projection surface 3.05 m × 2.06 m) using a single projector (Hitachi, Tokyo, Japan; CP-WX8255A; 1920 × 1080 High Definition) connected to the IRS computer. To produce the mirror effect, the projector is set in rear-projection mode. Notably, there are no technical limitations to project the avatar onto other projection devices such as a head-mounted display. Additionally, the avatar might be projected in 3D. In the current set-up, the avatar can be viewed in full-body size while the subject remains within an area of about 2.5 by 4 meters. The screen can be approached up to 1 meter. The size of the avatar is proportionally scaled with the distance as opposed to the screen. At a distance of about 2 meters, the avatar’s height is approximately 1.5 times smaller than the subject’s real height.

### Movement scaling

The movement scaling procedure is summarized in Figure 1. Movement scaling is programmed directly in D-Flow on the IRS using custom scripts. All rotation manipulations are performed in real-time using quaternions. Starting from the global position and rotation data of the MOCAP system, the data is first transformed into the D-Flow coordinate system. Starting from the root segment (pelvis), the hierarchy of the MOCAP skeleton is used to find the local rotation and position of all other segments. A reference rotation is acquired while the subject assumes a static base position. During movement the scaling is applied in the local space of each segment on the difference between the reference rotation and the current MOCAP rotation (updated during movement) using spherical linear interpolation (SLERP), or quaternion interpolation [24]. The SLERP operation returns a rotation interpolated between two rotations *q*_0_ and *q*_1_ according to an interpolation parameter (or scaling factor), *s*. For parameters *s* = 0 and *s* = 1 SLERP gives *q*_0_ and *q*_1_, respectively. In our case *q*_0_ is the reference rotation and *q*_1_ is the current MOCAP rotation. When for a given segment *s* < 1, SLERP returns an interpolated rotation that is a reduction of the current MOCAP rotation. For *s* > 1 the interpolated rotation is an augmentation of the current MOCAP rotation and follows the same direction. For *s* = 1 no scaling is applied and SLERP simply returns the current MOCAP rotation. Once the scaled rotation is applied locally on a segment, the positions and rotations of its child segments are updated according to this new scaled rotation. This process is performed upwards in the hierarchy up to the root segment (pelvis), resulting in a set of global rotations and positions that are applied onto the avatar. As such, both rotation amplitudes and velocities are scaled in real-time (total delay between movements and VR projection ranging between 90 and 120 ms). It is important to note that the scaling operation is performed locally on each segment and independently in each axis, so that in principle the scaling could be applied on any chosen segment depending on the required application.

### Scaling trunk movements

In this study, only the trunk, consisting of two segments (pelvis and thorax), was scaled in the sagittal plane (i.e. flexion-extension movements). Scaling factors ranged from *s* = 0.667 (corresponding to avatar movements being reduced 1.5 times) to *s* = 1.500 (corresponding to avatar movements being augmented 1.5 times). The range was determined empirically based on task performance in a two-alternative forced choice paradigm during pilot-testing in healthy subjects and in patients with chronic low back pain (for future clinical application). The two extremes (*s* = 0.667 and *s* = 1.500) produced movement scaling that could be clearly identified by the subject as being either reduced or augmented, and were used for familiarization and test trials. Two sets of five points equally spaced below and above *s* = 1 were used for analyses. As such, on a log scale, each point in the set below 1 had a corresponding inverse in the set above 1. The final set of scaling factors is listed in Table 2.

**Table 2:** **Scaling factors, and number of trials**

***s***	Log ***s***	Number of trials
0.667	−0.176	3 (test trials)
0.749	−0.126	4
0.793	−0.101	5
0.841	−0.075	6
0.891	−0.050	6
0.944	−0.025	7
1.000	0.000	7
1.060	0.025	7
1.123	0.050	6
1.190	0.075	6
1.261	0.101	5
1.336	0.126	4
1.500	0.176	3 (test trials)

## Proof of concept: perception of scaled trunk movements

### Subjects

The project was performed in collaboration with the Canadian Armed Forces. Healthy military subjects (aged between 18–55 years, men only to comply with the avatar’s gender) were recruited at a regional military base. Exclusion criteria included recurrent low back pain, low back pain that required medical care or that restricted work or recreation during the past 2 years, acute pain (pain score higher than 2/10, 0 = no pain, 10 = worst pain imaginable) at the time of testing, chronic pain (duration ≥ 3 months) during the last 6 months prior to participation, non-corrected visual impairments, repeated fractures, or other medical conditions (inflammatory, neurologic, degenerative, auto-immune, psychiatric) that could interfere with performance during testing. All assessments took place at the Centre Interdisciplinaire de Recherche en Réadaptation et Intégration Sociale of the Institut de réadaptation en déficience physique de Québec. The project was approved by the local institutional review board (#2013-323). All subjects received written and oral information, and signed informed consent prior to participation.

### Experimental procedure

#### Preparation

**Figure 2:**
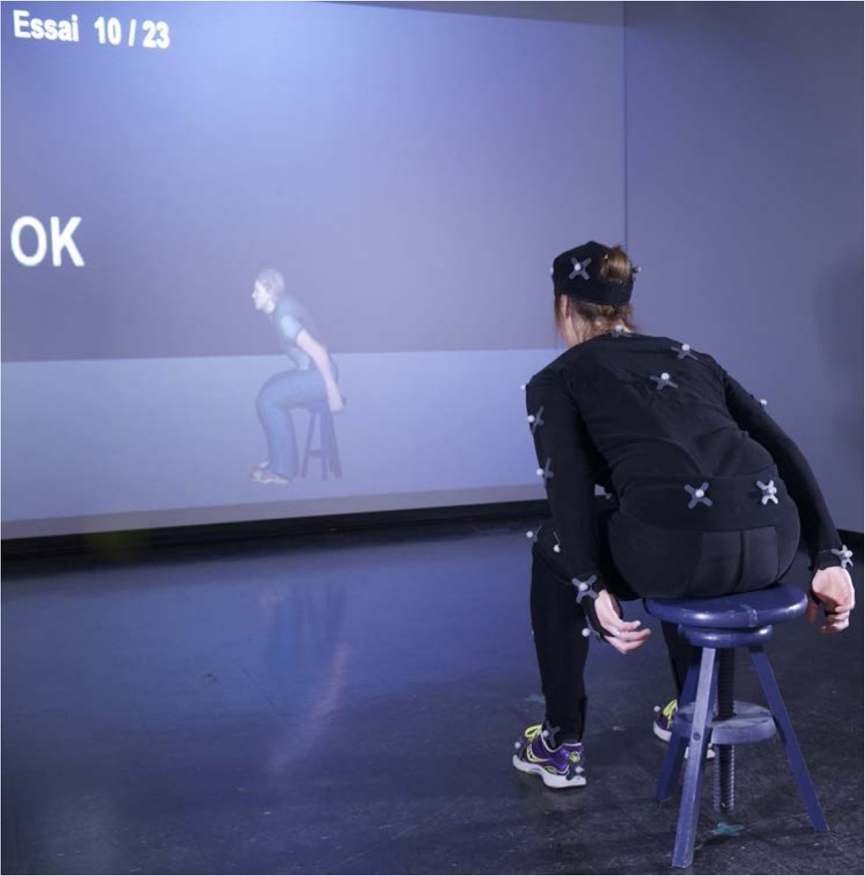
**Snapshot of the experimental procedure during a sitting block.** Each block consisted of 23 trials. The scaling factor was different for each trial. When subjects reached the required flexion angle (15°, 25° or 35°), simultaneous visual (OK) and auditory (bell-sound) feedback were provided. After returning to the base position, subjects had to decide whether the movements of the avatar were greater or smaller than their own movements (two-alternative forced choice paradigm).

Demographic and anthropomorphic data (weight, height, trunk height) were registered, after which the subject put on a body size-matched (4 sizes available) motion capture suit (OptiTrack, NaturalPoint, Corvallis, Oregon, USA) on which the markers were placed as described in the paragraph 'Motion capture system'. After calibrating the subjects for motion capture, they were placed in front of the projection screen (distance of 2 meters), the lights were dimmed, and the IRS software was activated to display the avatar in front of the subject (mirror mode, *s* = 1 = no modulation). A familiarization period including various pre-defined and spontaneous movements allowed subjects to explore the interaction with the avatar. Afterwards, the subjects remained seated facing the screen, but the avatar was medially rotated 90° so that it was displayed from the side (facing left) to allow for a better view of trunk flexion-extension movements (i.e. side-view mode). A snapshot of the experimental set-up is presented in Figure 2.

Subjects were instructed on static base positions for sitting and standing, which required them to keep their back and neck straight, their head facing the screen, arms falling naturally along the sides of the body, and feet aligned at shoulder width and pointing forward. For the sitting condition, subjects were placed on the stool that was adjusted to yield 90° of hip and knee flexion. For the standing condition, the subject was instructed to keep the knee joint partially flexed in order to maintain balance during trunk flexion. In the base position, the reference rotation was acquired and the trunk flexion angle was considered to be 0°. Subjects practiced the basics of the trunk flexion task in both positions while observing the simultaneous movements of the avatar on the screen (side-view, *s =* 1 = no modulation). The instructions were to move at a slow pace in one fluent movement towards a maximum angle of 35°, and this was demonstrated by the experimenter. Subjects received feedback on adherence to instructions.

#### Scaling task

The scaling task was introduced in the sitting position in 2 steps. First, the element of moving towards a predefined angle (unknown to the subject) was introduced (4 trials). The detection of these predefined angles by the IRS is described in detail under ‘Detecting and controlling flexion angles’. Subjects were required to start bending forward and, upon the appearance of the word “OK” on the screen along with a simultaneous bell-sound, return backwards to the base position. Second, a two-alternative forced choice paradigm was introduced (4 trials). After each trial, subjects had to decide whether the movements of the avatar were greater or smaller than their own movements. Subjects did not receive feedback on performance accuracy. After this brief training period, the experiment was started.

The number of experimental trials was weighted per scaling factor to acquire more data for relatively difficult trials involving small modulations, i.e. trials in which *s* was close to 1. The scaling factors were then distributed over 3 blocks of 23 trials each. The first 2 trials of each block were test trials (unknown to the subject), and were not further analyzed. The other scaling factors were distributed pseudo-randomly to ensure that blocks contained a balanced number of relatively easy and relatively difficult trials. As the tasks had to be performed while sitting and while standing, the total number of blocks was 6 (3 sitting, 3 standing blocks), and the total number of trials was 138. Sitting and standing blocks were alternated and the starting block (sitting or standing) was randomized across subjects. After each block there was a short break. After finishing all experimental blocks, the perceived interaction with the virtual mirror (immersion, distraction) was evaluated on a 1–7 scale using a selection of questions from the Presence Questionnaire (see Table 3 for the complete list of questions) [25]. The total duration of the experiment (including preparation) was about 2 h.

**Table 3:** **Virtual reality immersion and distraction (based on the Presence Questionnaire** [25]**)**

Questions		AV ± SD
Immersion	How much were you able to control the avatar (your virtual image)?	6.0 ± 0.7
	How responsive was the avatar to your movements?	5.8 ± 0.4
	How quickly did you adjust to the virtual environment experience?	6.2 ± 1.0
	How proficient in moving and interacting with the virtual environment did you feel at the end of the experience?	6.2 ± 0.8
	To what extent did the movements of the avatar seem natural to you?	5.1 ± 0.7
	How well could you examine the details of the avatar?	5.1 ± 1.1
Distraction	How much delay did you experience between your actions and the response of the system?	3.5 ± 2.0
	How much did the visual display quality interfere or distract you from performing assigned tasks or required activities?	1.8 ± 1.0
	How much did the control devices interfere with the performance of assigned tasks or with other activities?	1.3 ± 0.5

Scoring for immersion: 1 = not able/responsive/etc.; 7 = extremely able/responsive/etc. Scoring for distraction: 1 = no delay/interference; 7 = long delay/high interference.

#### Detecting and controlling flexion angles

Three predefined angles (15°, 25° and 35°) were programmed in the IRS to: 1) have subjects move within a safe range of motion (i.e. to avoid fatigue or pain) and 2) to introduce proprioceptive inter-trial-variability so that subjects would have to depend on both visual and proprioceptive feedback to perform the task correctly. The detection of flexion angles was based on the sagittal orientation of a vector connecting 2 markers on the back of the subject (C7 and T10). This orientation was considered to be 0° in the base position. When subjects reached the predefined angle for that trial, the IRS sent out the OK signal (screen) and simultaneous bell sound (audio), indicating to the subject to stop bending forward.

The 3 angles were distributed pseudo-randomly across the different blocks. Importantly, the 3 smallest scaling factors were not combined with a 15° detection angle, and the 3 largest scaling factors were not combined with a 35° detection angle. As such, the resulting avatar’s movements were also restricted to a limited range of motion. This avoided extremes in the visual feedback that would otherwise allow subjects to base their decision on visual feedback only. The important point from a methodological perspective was that subjects varied their flexion angles from trial to trial, and not that they achieved a specific flexion angle.

### Outcome parameters

For each individual subject, the responses to the two-alternative forced choice task were averaged over log-transformed scaling factors (see Table 2) and plotted (X = log-transformed scaling factor [−0.126; 0.126]; Y = percentage of trials for which the subjects responded that the avatar’s movements were greater than their actual movements [0; 1]). Then a sigmoid curve (Equation 1), with initial value X_Y0.50_ = 0, with constraints Y_MAX_ = 1 and Y_MIN_ = 0, and with a variable slope (*m*), was fitted to the data (Prism 6 for Windows, Graphpad Software Inc., La Jolla, CA, USA). From each curve, 3 data points were interpolated (X_Y0.25_, X_Y0.50_, X_Y0.75_), and used to determine the so-called point of subjective equivalence (PSE, Equation 2) and the just noticeable difference (JND, Equation 3). Theoretically, the chance distribution for a two-alternative forced choice paradigm predicts a PSE of 0, i.e. there is a 50% chance of responding “greater” or “smaller” when in fact no scaling has been applied. A PSE higher than 0 indicates that subjects tend to overestimate their own movements and a PSE lower than 0 indicates that subjects tend to underestimate their own movements. The higher the slope and the smaller the JND, the better subjects are able to discriminate between different levels of scaled feedback.
123

Task adherence was assessed by analyzing trunk movements for maximum flexion angles, maximum flexion velocity and for the fluency of movement around the maximum flexion angle (number of zero-crossings in trunk acceleration between the maximum flexion and maximum extension velocity) for each of the predefined flexion angles (15°, 25°, 35°), using in-house scripts written in Matlab (version R2010b, The Mathworks Inc., Natik, MA, USA). Data was filtered using a second-order double pass Butterworth filter (4 Hz). Trunk movement analyses were performed based on 3 markers located on the back of the subject (C7, T10 and scapula), and focused on the sagittal plane only.

### Data analysis

For each of the outcome parameters (X_Y0.25_, PSE, X_Y0.75_, JND, *m*) the normality of the data distribution (the skewness of the distribution) and presence of outliers (data outside 1.5 times the interquartile range) was assessed and descriptive statistics were calculated (IBM SPSS for Windows, version 22.0.0.0, USA). Movement data was analyzed using multivariate tests with within-subject factor [Angle] (15°, 25°, 35°). Data is presented in text as mean ± standard deviation.

## Results

A total of 11 healthy subjects participated in the experiment. One subject showed poor task adherence and was additionally identified as an outlier based on psychophysical curve metrics and movement data. As such this subject was excluded from the analyses. The final sample therefore consisted of 10 male subjects, having a mean age of 28 ± 5 years (range: 22–37), weight of 88 ± 14 kg (range: 62–108), height of 176 ± 10 cm (range: 165–201), and Body Mass Index (BMI) of 28 ± 4 (range: 23–34).

### Two-alternative forced choice paradigm and psychophysical curve

**Figure 3:**
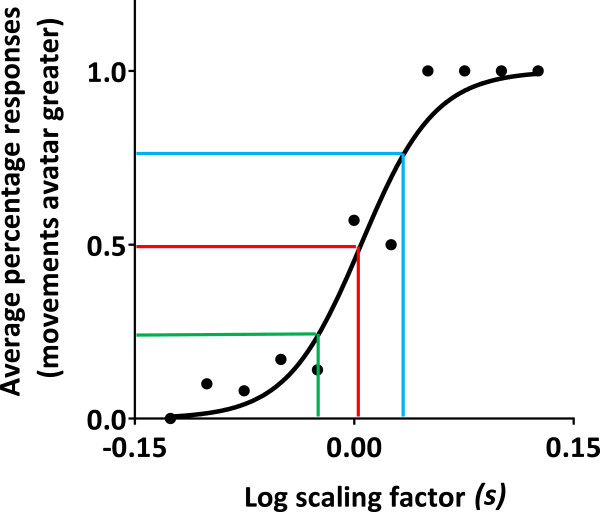
**Average response frequencies (black dots) and curve fitting results (black line) for a representative subject.** For log scaling factors smaller than 0, avatar movements were reduced. For log scaling factors greater than 0, avatar movements were augmented. For a log scaling factor of 0, no modulation was applied. Colored lines indicate the curve metrics derived: X_Y0.25_ (green), X_Y0.50_ = point of subjective equality (PSE) (red), and X_Y0.75_ (blue). The PSE was close to 0 consistent with expectations for a two-alternative forced choice paradigm.

**Figure 4:**
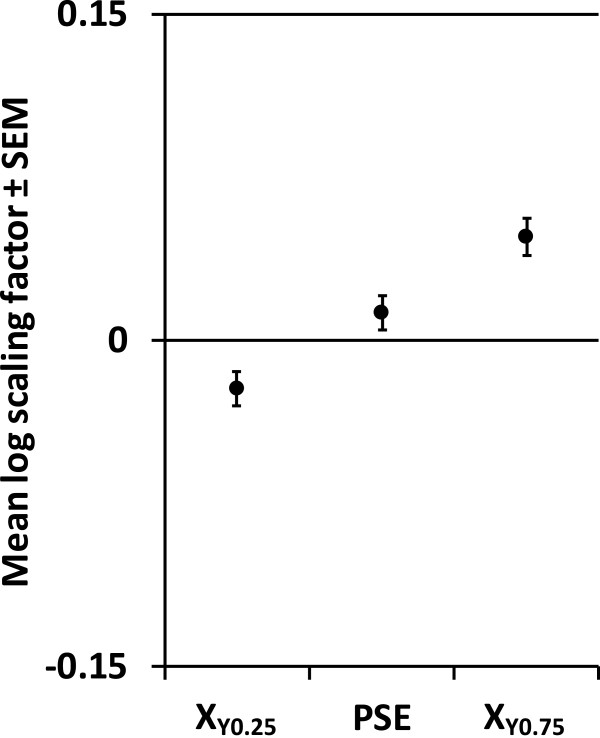
**Curve metrics as derived from individual curves (n = 10), mean ± SEM.** X_Y0.25_: interpolated log scaling factor at a response frequency of 0.25; X_Y0.75_: interpolated log scaling factor at a response frequency of 0.75; PSE: point of subjective equality.

The data followed a normal distribution (i.e. skewness values close to 0). Figure 3 presents the data and curve fitting results for a representative subject. In general, the goodness of fit for these individually fitted curves was high (R^2^ range: 0.89 - 0.98). Group averaged interpolated X_Y0.25_, PSE, and X_Y0.75_ (± SEM) are presented in Figure 4. The 95% confidence interval for the PSE ranged from −0.003 to 0.028, indicating that the PSE was not significantly different from 0. The average JND was 0.035 ± 0.007 (range 0.026 - 0.042), the average curve slope *m* was 14.1 ± 2.7 (range 10.1 - 18.6), and the average percentage of correct responses was 83% ± 4% (range 60% - 100%).

### Virtual reality immersion

Table 3 presents the average scores relating to the subjective experience with the virtual mirror. Despite perceived delays, and despite the fact that the “fit” of the avatar (i.e. regarding anthropomorphic characteristics) was better for some subjects than for others, immersion was found to be relatively good. Distraction due to visual display quality and/or the control devices (e.g. Vicon suit and markers) were considered minor and did not interfere or interfered only somewhat with task performance.

### Task adherence (movement kinematics)

The data followed a normal distribution (i.e. skewness values close to 0). Movement analyses revealed distinct maximum flexion angles for each of the predefined angles (15°: 32° ± 5°; 25°: 38° ± 4°; 35°: 43° ± 4°)(F_2,8_ = 49.603, p < 0.001). As expected, maximum flexion angles were higher than the predefined angles due to the subjects’ reaction times following the appearance of the “OK” sign and simultaneous bell-sound. Importantly, subjects varied their movement angles from trial to trial as required. Flexion movement velocity (15°: 31° ± 9°/s; 25°: 31° ± 8°/s; 35°: 31° ± 8°/s) was comparable for each of the angles (F_2,8_ = 1.506, p = 0.279). As expected, movement fluency (15°: 0.9 ± 0.5 zero-crossings; 25°: 1.0 ± 0.4 zero-crossings; 35°: 1.3 ± 0.5 zero-crossings) was slightly better for smaller angles (F_2,8_ = 5.725, p = 0.029).

## Discussion

A virtual mirror allowing for the scaling of visual movement feedback was developed by integrating motion capture, virtual reality and projection systems. In a proof-of-concept study in healthy adults, the ability to detect scaled feedback on trunk flexion movements was according to expectation. Performance could be modelled using a sigmoid curve with a high goodness of fit, and confirmed unbiased perception (PSE not different form 0) and high discriminative ability (small JND) in healthy adults.

### Virtual mirror components and performance

The real-time full-body virtual mirror that was developed in this study displayed a realistic full-body avatar that responded to full-body movements in all movement planes in real-time, and allowed for the real-time scaling of visual feedback on movements at a given joint. As such, the developed virtual mirror extends existing VR systems in motor and pain rehabilitation (e.g. [10–12, 3, 13, 15]) and further enables the use of realistic full-body avatars.

Throughout the test-phase, the real-time performance of the virtual mirror was stable, and the IRS refresh rate was according to specifications (60 Hz). Although the total delay between actual movements and projected movements was found to be between 90 and 120 ms, perceptual delays were reported ranging from “no delays” to “long delays”. Upon verification, the computational load associated with the scaling of movements could be ruled out as a potential source of delay, suggesting the total delay was mainly caused by delays in the communication between the MOCAP system and IRS. Together with the IRS supplier, we are currently trying to further improve the communication between the two systems.

Due to the exploratory nature of the study, initially only one realistic male avatar model was implemented. As expected, the “fit” of this “one-size fits all”-avatar was better in some subjects than in others. This might be improved by incorporating additional anthropomorphic data, such as BMI, into the avatar rigging process. However, regardless of perceived delays and avatar “fit” issues, immersion was reported to be good. As such, we are confident that the virtual mirror worked sufficiently well to apply scaled feedback and to assess the ability to perceive this scaled feedback in healthy subjects. This is further substantiated by the relatively small between-subject variability observed in this proof-of-concept study.

### Detecting scaled feedback

Movement kinematics were consistent with instructions to move slowly and in one fluent movement towards a set of predefined flexion angles, confirming task adherence. As such, both proprioceptive (real flexion angles) and visual feedback (avatar flexion angles) varied from trial to trial. Together, this suggests that subjects relied on a combination of visual and proprioceptive feedback to perform the task and seems consistent with the spontaneous reports of subjects perceiving the two-alternative forced-choice paradigm as being difficult.

Using the current virtual mirror set-up, the detection of scaled feedback could be modelled using a sigmoid curve with a high goodness of fit. Importantly, the subjects’ performance was consistent with expectations for a two-alternative forced choice paradigm, i.e. the PSE being not significantly different from 0 reflecting unbiased perception, and small JNDs reflecting high discriminative ability. Back-transforming the average JND to a linear scale reveals that avatar movements had to be augmented or reduced only 1.07 times to be accurately detected, i.e. staying within the range of 2 scaling levels below or above 1. In addition, the between-subject variability of psychophysical outcome parameters appears to be sufficiently small to allow for distinguishing normal from abnormal responses in future studies (e.g. as compared to clinical populations). Together, these results confirm the validity of our method to assess body perception during active trunk movement.

Some methodological limitations need to be considered, including the relatively small number of subjects, the relatively low number of trials (divided over sitting and standing blocks), and the application of scaling in one plane of movement using a relatively small range of motion (the maximum predefined angle being 35°). Additionally, the experimental task required a mental rotation (due to presentation of the avatar in side-view mode) which could have impacted on task performance. However, despite these limitations, the between-subject variability was relatively small and immersion was reported to be good. Additional study of protocol parameters (e.g. optimal number of trials/blocks), and complex analyses of curve metrics and movements (e.g. to distinguish different movement strategies) may help to further improve the protocol and the interpretation of results.

### Potential applications

To date, our scaling protocol using the virtual mirror was implemented for trunk flexion only (involving the pelvis and trunk segments). In principle, scaling could be applied on any chosen body segment to fit the required application. In the near future, we envision two main applications.

First, the virtual mirror might be used as an assessment tool, i.e. to assess body perception during active movement. This would extend currently available assessment tools that commonly assess body perception under static conditions. Likewise, this application would allow for the assessment of body perception disturbances in patients, which may inform clinical management. The present protocol for trunk flexion was developed for the assessment of body perception in patients with chronic low back pain, in whom body perception disturbances and fear of movement are thought to play an important role in disease pathology [26–28]. Other relevant patient populations for which similar pathophysiological mechanisms have been proposed include patients with complex regional pain syndrome, fibromyalgia, or phantom-limb pain [23, 29–31].

Second, the virtual mirror might be used as an intervention tool, alone or in combination with other cognitive or physical interventions. As introduced in the background section, prolonged periods of scaled feedback might be applied to promote or prevent specific movements in patients displaying altered perceptions of body movements [17, 18]. Additionally, the virtual mirror could be used to overcome fear of movement (kinesiophobia) in patients with chronic pain by providing an extension to, or by replacing, graded in-vivo exposure therapies [32, 33], such as recommended for the treatment of patients with chronic low back pain displaying high levels of kinesiophobia [34].

## Conclusions

The new virtual mirror extends existing VR systems for motor and pain rehabilitation by providing scaled feedback, and enables the use of realistic full-body avatars. After having demonstrated proof-of-concept in healthy adults, we are now exploring the current virtual mirror set-up as a tool to assess body perception disturbances in patients with chronic low back pain.

## Abbreviations

BMI: Body mass index
IRS: Interaction and rendering system
MOCAP: Motion capture
SLERP: Spherical linear interpolation
VR: Virtual reality.
